# Laparoscopic Versus Robot-Assisted Pyeloplasty in Adults—A Single-Center Experience

**DOI:** 10.3390/jpm12101586

**Published:** 2022-09-26

**Authors:** Orel Carmona, Zohar A. Dotan, Miki Haifler, Barak Rosenzweig, Dorit E. Zilberman

**Affiliations:** Department of Urology, Chaim Sheba Medical Center, Tel Hashomer, Israel, Affiliated to the Sackler School of Medicine, Tel-Aviv University, Tel-Aviv 6997801, Israel

**Keywords:** laparoscopy, robotic surgery, pyeloplasty, uretero-pelvic junction obstruction

## Abstract

***Background***: Laparoscopic (LP) and robot-assisted pyeloplasty (RAP) are minimally invasive techniques for correcting uretero-pelvic junction obstruction (UPJO). We retrospectively compared the clinical outcomes of all adults who underwent RAP (*n* = 41) to those who underwent LP (*n* = 24) for UPJO at our institution between 2003–2022. ***Methods***: Age, sex, body mass index, surgical side, past abdominal/endoscopic surgeries, pre- and postoperative renal scans, pre- and postoperative serum creatinine levels, operative time (OT), presence of crossing vessels, estimated blood loss, postoperative complications, length of hospital stay, time to JJ stent removal, follow-up length, and postoperative hydronephrosis were analyzed. ***Results***: The groups were demographically comparable. The mean total and skin-to-skin OTs (minutes) were significantly longer in the RAP group than in the LP group (242.4 ± 55 vs. 161.4 ± 40 *p* < 0.001; 163.7 ± 41.8 vs. 124.3 ± 30.3 *p* = 0.006, respectively). Hospital stay (days) was shorter in the RAP group (3.3 ± 2.1 vs. 7.3 ± 2.5 *p* < 0.001). Postoperative complication rates were identical for both groups. The LP group had a significantly longer follow-up period (85.2 ± 73 vs. 19 ± 14 months *p* < 0.001). The success rates for the LP and RAP groups were 87.5% and 90.6% (*p* = 0.708). ***Conclusions***: RAP achieves equivalent results to LP, in adult patients. A longer OT may be expected with the robotic system since it can handle more complicated cases.

## 1. Introduction

Congenital or acquired uretero-pelvic junction obstruction (UPJO) is a result of obstructive stenosis between the renal pelvis and the proximal ureter. The obstruction interrupts urinary flow from the renal pelvis into the ureter, the renal pelvis becomes dilated, the renal parenchyma is lost, and kidney function deteriorates [1]. The causes of UPJO include an aperistaltic ureteral segment; a crossing vessel to the kidney’s lower pole; ischemic or inflammatory strictures; obstructing nephrolithiasis; adhesions; malignancies; fibroepithelial polyps; and more [1]. Possible complications of UPJO include recurrent infections, recurrent renal calculi formation, renal colic, and the deterioration of renal function.

An open retroperitoneal approach (pyeloplasty) was first described by Trendelenburg in 1886, and the first successful surgery was performed by Kuster 5 years later [1].

In 1949, Anderson and Hynes presented the principles of an open retroperitoneal approach pyeloplasty [2]. Those principles were widely adopted worldwide and became the most common surgical technique for the correction of UPJO.

Laparoscopic pyeloplasty (LP), which was performed according to the Anderson–Hynes principles, was first described in 1993 by Schuessler et al. [3] and Kavoussi and Peters [4], who reported similar success rates compared to the open approach and with shorter recovery time and better cosmetic results.

Following the introduction of the da Vinci robotic surgical system in late 2000, an increasing number of surgeons have been taking advantage of three-dimensional imaging, six degrees of freedom, and the elimination of tremor provided by the system for enhanced performance of complicated surgeries.

In 2002, Gettman et al. [5] were the first to describe the first case series of nine robotic-assisted pyeloplasties (RAP), and they attributed the high success rates almost entirely to the technical advantages provided by the innovative da Vinci computer-enhanced system.

The high costs of implementing the robotic system, however, have been of major concern among some medical centers, raising the question of economic viability. For example, the average cost of laparoscopic surgery at our medical center as of 2021 was 1550 USD, while the average cost of robotic surgery was 3800 USD.

The English literature is replete with studies that sought to compare the outcomes of RAP to those of LP or to an open approach. Unfortunately, the vast majority of those studies are either low-quality, missing data, or combine pediatric and adult populations in their statistical analysis. In the present study, we aimed to compare the clinical outcomes of RAP to those of LP performed solely in an adult population at a single medical center.

## 2. Materials and Methods

Interventionary studies involving animals or humans, and other studies that require ethical approval, must list the authority that provided approval and the corresponding ethical approval code. Following approval by the Chaim Sheba Medical Center Institutional Review Board (#SMC-20-7759), we retrospectively collected the data of all adult patients (≥18 years of age) who had undergone either first-time LP or RAP for UPJO at our institution between 2003–2022. Specifically, LP had been performed between 2003–2008 by one surgeon, while RAP had been performed from 2015 onward by another surgeon. Both surgeons had at least 5 years of experience in laparoscopic and robotic surgeries, respectively, at the date of first recording for each. Only a few of those surgeries took place between 2008 and 2015 due to human resource issues, and those that were performed had been carried out in an open retroperitoneal approach and by various surgeons.

The indications for pyeloplasty in the adult population were as follows: persistent pain, obstruction displayed on renal scintigraphy, and recurrent urinary tract infection, all in association with hydronephrosis due to UPJO as demonstrated by computerized tomography.

### 2.1. Operative Technique

**Figure 1:**
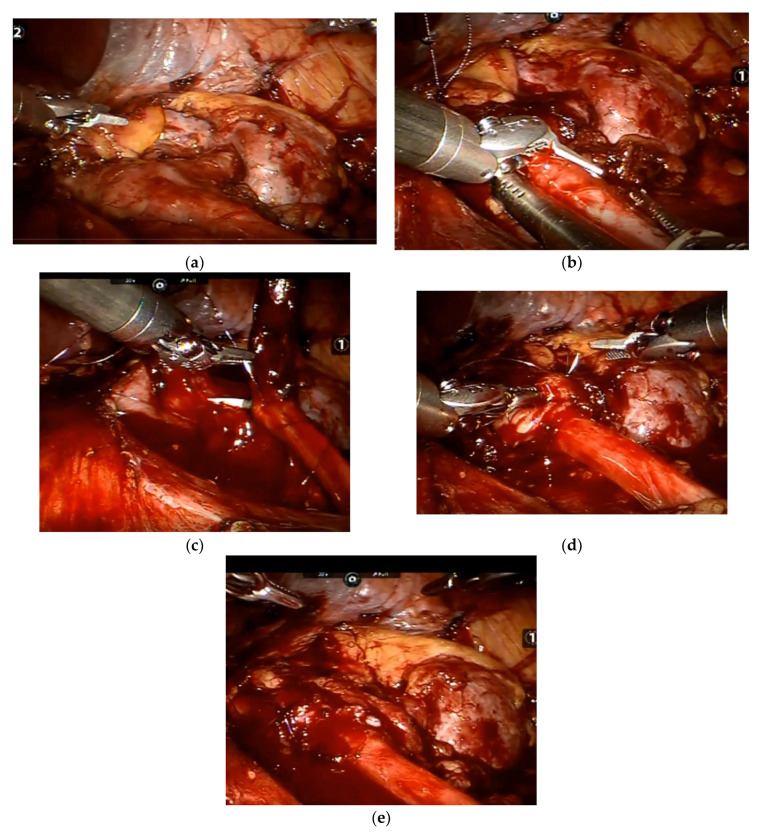
Selected stages during robot-assisted pyeloplasty: (**a**) UPJ exposure; (**b**) ureteral spatulation in the lateral aspect after resecting the sick UPJ portion; (**c**) uretero-pelvis anastomosis in the posterior aspect over a ureteral JJ stent; (**d**) uretero-pelvis anastomosis in the anterior aspect; and (**e**) at the procedure’s conclusion.

Both LP and RAP were performed in a transperitoneal approach and as previously described by others [6]. RAP surgeries had been performed using the da Vinci Si robotic system (Intuitive, Sunnyvale, CA, USA). Essentially, after placing the patient in a lateral decubitus position and following abdominal insufflation using CO2 gas, three working ports were placed: one was a 12 mm camera port at the lateral border of the rectus muscle in line with the umbilicus, and the other two were 8 mm robotic ports in the upper and lower abdominal quadrants, respectively, placed 8–10 cm cranial and caudal to the camera port. In right-sided cases, a subxyphoidal 5 mm port was placed for liver retraction. The surgical stages included reflection of the white line of Toldt, exposure of the UPJ area, resection of the sick portion, proximal ureteral spatulation, and renal pelvis reduction wherever applicable. In cases of an associated pyelolithotomy, a flexible cystoscope (11272CU1 Karl Storz, Tuttlingen, Germany) was introduced into the detached renal pelvis. The entire collecting system was carefully scoped for the presence of stones, which were removed with a designated endoscopic basket (12 mm, 1.9Fr zero-tip stone retrieval basket, Boston Scientific, Marlborough, MA, USA). Subsequently, a JJ stent was placed in the ureter in an antegrade fashion over a guidewire, followed by an UPJ anastomosis starting from the dependent portion of both the ureter and renal pelvis, suturing their posterior walls over the stent and concluding with closure of their anterior walls. A 7 Fr Jackson–Pratt drain was left in place to monitor urinary leakage or unexpected postoperative bleeding. Selected surgical stages during RAP are described in Figure 1. The JJ ureteral stent was removed 4–6 weeks following the operation.

### 2.2. Data Collection and Definition of Variables

Data on the following parameters were recorded and analyzed: age, sex, body mass index, surgical side, previous abdominal surgeries, previous endoscopic surgeries (i.e., stone fragmentation, and endopyelotomy), preoperative renal scintigraphy findings, serum creatinine level, total and skin-to-skin operative times, the presence of a crossing vessel, estimated blood loss, postoperative complications, length of hospital stay, time to JJ stent removal, follow-up length, the presence of hydronephrosis, post-operative renal scintigraphy findings, and the creatinine level at the last follow-up. Short-term postoperative complications were classified according to the Clavien–Dindo classification system [7].

We defined total operative time as the time that had elapsed between the patient’s entering the operating theatre until transfer to the recovery room. Similarly, skin-to-skin operative time was calculated as the time since the first skin incision to the closure of the last incision.

Surgical success was defined by not needing a second surgery due to re-stenosis or if there had been an improvement in the extent of hydronephrosis as seen on images performed during the follow-up period.

### 2.3. Statistical Analysis

Continuous variables were presented as mean ± standard deviation (SD), and categorical variables were presented as the number and percentage of individuals in each group. Categorical variables were compared by the Chi-squared test, while *T*-tests were used to examine differences in means of continuous variables. *p* values <0.05 were considered significant. Statistical analyses were performed with IBM SPSS software, version 26.0 (Armonk, NY, USA: IBM Corp, 2019).

## 3. Results

A total of 24 patients underwent LP, and 41 patients underwent RAP for UPJO during the study period. Their demographic, surgical, and postoperative data are detailed in Table 1.

The two groups did not differ demographically. While the skin-to-skin operative time was significantly longer for the RAP group compared to the LP group, both the length of stay and the follow-up duration were significantly shorter.

Four patients who underwent LP had previously undergone abdominal surgeries consisting of endoscopic procedures for stone fragmentation, and two of them (8.3%) underwent UPJO treatment (i.e., uretero-nephroscopy with endopyelotomy and laser lithotripsy).

Eleven patients in the RAP group had undergone previous abdominal surgeries that included endoscopic procedures in five (12.1%) of them. Four patients from the LP group (16.6%) and seven patients from the RAP group (17%) also underwent pyelolithotomy during the index surgery. One LP patient (4.1%) underwent conversion to open surgery due to difficult anastomosis.

There were no significant differences in the early (up to 1 month) postoperative complications sustained by both surgical groups (Table 2).

Complete stone retrieval failed in two patients who underwent LP, and a second stone fragmentation procedure was later required for both.

Three patients in the LP group (12.5%) required a second surgery due to re-stenosis and persistent hydronephrosis. One patient in the RAP group (2.4%) underwent diagnostic ureteroscopy 4 months following the index surgery due to persistent pain and evidence of hydronephrosis and retained stone on sonographic images. A 3 mm stone retrieval was performed in that case.

Of the 32 RAP patients for whom long-term follow-up was available, 19 (59.4%) had evidence of hydronephrosis on imaging; however, the postoperative extent of hydronephrosis improved compared to the preoperative level in 16 of them. We do not have information on the long-term follow-up hydronephrosis status in the other three patients. The overall estimated success rate for each technique was calculated at 87.5% for the LP group (21 out of 24) and 90.6% for the RAP group (29 out of 32) (*p* = 0.708).

## 4. Discussion

Open pyeloplasty has long been considered the definitive surgical approach for the correction of UPJO, with an estimated 90–100% success rate [8]. Long recovery times, the frequent use of analgesics, and the consequent long hospital stays, however, have motivated the search for an alternative approach that will decrease time to recovery on the one hand while achieving the same success rates as open surgery on the other hand.

The widespread introduction of laparoscopic surgery during the 1990s inevitably involved the urologic arena as well, dramatically decreasing both the length of hospital stay as well as the patient recovery time while achieving enhanced cosmetic results.

Notwithstanding, there has been much disappointment and frustration stemming from complicated maneuvers, such as suturing, resulting in a steep learning curve, whereupon laparoscopic surgery became a much longer and more exhaustive option compared to the open approach [6]. In the hands of highly skilled surgeons, the overall success rates of LP have been estimated at 95–98% [8]; however, the time to achieve such a high level of training and experience has left such outstanding success rates limited to relatively few surgeons.

The introduction of the da Vinci robotic system brought an exponential increase in pyeloplasties worldwide [9]. The underlying reason for its spiraling popularity was the system’s Endowrist technique, which could precisely translate the surgeon’s maneuvers within the robotic console into robotic arm movements within the patient’s anatomy. This led to a vast improvement in the ability of surgical reconstruction that involved complicated suturing, with a level of precision that equaled or exceeded the one observed in the open approach.

There is an abundance of studies in the English literature that compare the various pyeloplasty techniques and approaches; however, most of them include combined adult and pediatric populations. Our extensive search of the literature yielded only five studies on exclusively adult populations (i.e., >18 years of age) and that compared LP and RAP approaches [6,10,11,12,13]. Their findings are summarized in Table 3.

Our current patients’ demographic data resemble those presented in the other case series: they were in their 4th–5th decades of life, they had no laterality dominance, the rate of the estimated blood loss during their surgeries was low, and the overall complication rate was low as well. An interesting finding in all of the five adult case series, including the present one, is the relatively high incidence of a crossing vessel to the renal lower pole in the UPJO cases (~50%) compared to the general population (~30%) [1]. It has been suggested that there are indolent crossing vessels that do not create any response in the UPJ area [1]. The pathologic basis of UPJO is an aperistaltic segment resulting from fibrosis and obstruction.

Krajewski et al. found no pathological histology in many patients with a crossing vessel to the lower pole and observed that crossing vessels only created mechanical obstruction [1]. In other patients, the crossing vessels caused inflammatory response in the UPJ area; fibrosis; and smooth-muscle hypertrophy that, in turn, led to obstruction [1]. Those authors proposed that this diversity is the reason why the role of crossing vessels in UPJO pathophysiology remains controversial [1].

The findings of the present study differ from earlier ones in three parameters: (1) the longer length of stay of the LP cases, (2) the longer total and skin-to-skin operative time in the RAP group compared to the LP group, and (3) the longer overall follow-up time in general and in the LP group in particular. The first finding can be explained by the very stringent protocol that has been followed in our department during the laparoscopic era. Longer catheter and drainage times were the standard of care due to uncertainty about the suturing quality. Later on, with the establishment of high success rates, this draconian protocol was abandoned.

Similar to Link et al. [11], we have been witnessing a longer total and skin-to-skin operative time in the RAP group than in the LP group. Our medical center has no permanent team in the operating theatre dedicated to robotic urological surgeries, and we believe that its absence contributes to prolonging the time to room configuration, patient placement, equipment preparation, etc. Another possible reason is the complexity of the cases, which appears to increase over time.

The main limitation of the present study is its retrospective design. While the study groups are relatively small, pyeloplasty in the adult population is not a common surgical procedure and is one that is indicated in very selected cases compared to the pediatric population. The main advantages of our study are its 20-year perspective design (2003–2022) and its relatively long postoperative follow-up period, which is not available in other studies.

We consider that the continuous presence of hydronephrosis observed in adults during long-term follow-up is not necessarily a surgical failure but rather that it reflects a chronic state that is not associated with persistent obstruction. This notion is supported by our patients’ postoperative renal nuclear scans, which were not obstructive in most cases. Moreover, we believe that RAP is as good as LP, and that the decision regarding technique selection should be in accordance with the surgeon’s discretion and skills. Notwithstanding, the advantages of better visualization and the precision of movement that significantly reduce the level of complexity and that are made possible by the da Vinci robotic surgical system are highly attractive to a tech-savvy surgeon.

## 5. Conclusions

RAP achieves the same results as LP among adults with UPJO, but it is superior to LP because it provides the surgeon with an overall better surgical experience. More complicated cases can be carried out with the da Vinci robotic system than with both open surgery and LP, but some lengthening in operative time may be expected.

## Figures and Tables

**Table 1:** Demographic, surgical, and postoperative data of the study participants according to surgical approach.

Variable	Laparoscopic Pyeloplasty (*n* = 24)	Robotic Pyeloplasty (*n* = 41)	*p*-Value
	Demography		
Age (years)	40 ± 17	36 ± 13	0.297
Sex Male Female	14 (58.3%)	20 (48.7%)	0.608
	10 (41.7%)	21 (51.3%)	
Surgical side Right Left	17 (70.8%)	22 (53.6%)	0.199
	7 (29.2%)	19 (46.4%)	
Body mass index	28.1 ± 8.14	25.3 ± 4.9	0.164
Preoperative kidney function assessment per nuclear renogram (%)	38 ± 13.9	41.3 ± 11.4	0.34
Preoperative creatinine level (mg/dL)	1.09 ± 0.23	0.9 ± 0.24	0.109
	**Surgical Data**		
Total operative time (min)	161.4 ± 40	242.4 ± 55	**<0.001**
Skin-to-skin operative time (min)	124.3 ± 30.3	163.7 ± 41.8	**0.006**
Crossing vessel	8 (33.3%)	22 (53.6%)	0.13
Estimated blood loss (mL)	36 ± 90	50 ± 144	0.784
Length of hospital stay (days)	7.3 ± 2.5	3.3 ± 2.1	**<0.001**
	**Post-Operative Data**		
JJ stent removal (days)	42 ± 7	38 ± 7	0.580
Follow-up length (months)	85.2 ± 73	19 ± 14	**<0.001**
Postoperative kidney function assessment per nuclear renogram (%)	35 ± 11.1	42.6 ± 10.9	0.281
Creatinine level at last follow-up (mg/dL)	0.81 ± 0.22	0.86 ± 0.28	0.611

Values are given as mean ± standard deviation or *n* (%). **Bold** indicates significant.

**Table 2:** Early postoperative complications according to surgical approach.

*Complication*	*Clavien–Dindo* *Classification*	*Laparoscopic Pyeloplasty* *(N)*	*Robot-Assisted Pyeloplasty* *(N)*
*Anastomotic leak with renal drainage*	*IIIA*	*2*	*2*
*Anastomotic leak without renal drainage*	*I*	*2*	*4*
*Hematuria*	*II*	*1*	*1*
*Urinary infection*	*II*	*1*	*2*

**Table 3:** Comparisons between laparoscopic pyeloplasty and robot-assisted pyeloplasty in the adult population, as reported in the literature.

First Author	Period	Single Surgeon	Origin	Surgery	No. of Patients	Age (yr, Mean or Rrange)	Male	Female	Side Right	Left	BMI (Mean or Range)	Crossing Vessel (%)	Operative Time (Min)	EBL (mL)	Hospital Stay (Days)	Success Rates (%)	Follow-Up Length (Months)	Complications (%)
Bernie J.E., 2005 [10]	1999–2003	Yes	USA	LP	7	34 (18–55)					24 (19–31)	86	312 (240–390)	40 (5–200)	3 (2–4)		10 (range 5–15)	28.5
				RAP	7	32 (25–49)					26 (21–32)	57	324 (252–420)	60 (50–100)	2.5 (2–6)		24 (range 22–30)	28.5
Link R.E., 2006 [11]	2004	Yes	USA	LP	10	38 ± 14	4	6	4	6	24.3 ± 14.5		134.8 ± 20.6 *	no difference			5.6 ± 2.2	
				RAP	10	46.5 ± 16.9	3	7	5	5	23 ± 5.9		173.8 ± 15.4 *	no difference				10
Bird V.G., 2011 [12]	1999–2009	No	USA	LP	74	39.8 ± 13.9	35	39	46	27	26 ± 5.6	51.4	187 ± 69	<50	2.5	95		2.7
				RAP	98	39.6 ± 15.2	46	52	58	40	25.7 ± 5.8	63.3	189 ± 62	<50	2.5	93.4		5.1
Pahwa M., 2014 [13]	2011–2013	Yes	India	LP	30	34.4 (23–39)	13	17	15	15	25.5 (20–30)	33.3	191.6 (145–276) *	55 (35–120) *	3 *	97	18 (8–23)	11.4
				RAP	30	32 (19–49)	12	18	14	16	26.4 (19–30)	33.3	141.7 (110–235) *	46.4 (30–95) *	2.45 *	97	13.5 (5–20)	8
Rasool S., 2020 [6]	2015–2018	N/A	India	LP	34	31 ± 14	18	16	18	16	24.1 ± 2.9	29.4	187.8 ± 22.1	45.6 ± 20.3	2.82 ± 1.18	94.1	6	9
				RAP	34	29.3 ± 11.1	24	10	14	20	25.1 ± 2.7	29.4	136.7 ± 25.1	42.9 ± 20.8	2.65 ± 1.22	97	6	6

LP, laparoscopic pyeloplasty; RAP, robot-assisted pyeloplasty; BMI, body mass index, and EBL, estimated blood loss. * Statistically significant.

## Data Availability

Restrictions apply to the availability of these data. Data were obtained from the Chaim Sheba Medical Center records database and are available upon request.
